# Using Simulation-Based Methods to Support Demonstration of Competencies Required by Micro-Credential Courses

**DOI:** 10.7759/cureus.16908

**Published:** 2021-08-05

**Authors:** Eva H Peisachovich, Adam Dubrowski, Celina Da Silva, Bill Kapralos, Jennifer E Klein, Zipora Rahmanov

**Affiliations:** 1 Medical Education and Simulation, York University, Toronto, CAN; 2 Research and Development, Ontario Tech University, Oshawa, CAN; 3 Software Informatics Research Centre, Ontario Tech University, Oshawa, CAN; 4 School of Nursing, York University, Toronto, CAN

**Keywords:** remote learning, simulation, competencies, micro-credential courses, health professionals education

## Abstract

The rise of the digital revolution has disrupted entire industries and job markets, leading individuals to either upgrade or transfer their skills in order to continue within their designated fields or transition to new workplace contexts. Employers expect their employees to apply their knowledge to real-world settings, analyze and solve problems, connect choices to actions, and innovate and create. Moreover, the COVID-19 pandemic has exacerbated changes to the educational landscape by forcing online and remote contexts; physical distancing and other preventive measures have necessitated a shift towards increasing the use of disruptive digital technologies- extended reality (e.g., virtual and augmented reality), gaming, and additive manufacturing-in simulation delivery. Yet Canada’s economic and demographic data suggests that many new graduates struggle to transition from school to working life. The confluence of these factors has led to a need for both individuals and higher education institutions to upgrade and adapt to new digital techniques and modalities. As these needs grow, simulation-based education (SBE) techniques and technologies-already an integral part of training for some professions, including nursing, medicine, and various other health professions-are increasingly being used in digital contexts.

In this editorial, we provide our perspective of the socio-technological movement associated with health-professions education (HPE) within the SBE context and examine the application and implementation of micro-credentialing within this field. We also discuss the various levels of expertise that learners may acquire. From this vantage point, we address how SBE can complement the assessment of competencies that learners must demonstrate to attain micro-credentials and explore micro-credentialing’s advantages for, and use in, HPE.

## Editorial

Health-professions education and the simulation context

A large body of literature shows that simulation-based education (SBE) has been reliably used in health-professions education (HPE) to teach, assess, and measure competencies [1]. SBE provides a safe framework in which learners can make mistakes without causing harm (as may occur in real-life situations) and an opportunity to reflect on the action, both in action and beyond action, to learn from these errors. Moreover, SBE affords trainees an opportunity to experience and participate in situations that are rare in practice.

Simulation is now becoming more technologically advanced; while traditional bench-top, high-fidelity mannequins, and in-person simulation are still used, technology has allowed for digital simulation that extends beyond the realm of in-person experiential learning. The recent pandemic is accelerating these changes to the landscape of simulation practice. For example, the use of Simulated Persons (SPs)-an essential tool for developing soft skills such as communication, creativity, contextual behavior, motivation, self-efficacy, empathy, emotional intelligence, and interpersonal skills-has shifted to a virtual delivery setting, supporting both the shift to online and the virtual interactions and the increased need for telecommunication and teleconsultation.

As required skills in the field evolve, so too does the technology required to teach them. Virtual patients are now commonly used at the undergraduate level and have been shown to create effective learning opportunities or scenarios that enhance the learners’ perceived self-efficacy and confidence in their communication skills. Extended reality-based applications are now also being used in SBE. Extended reality (XR) is an umbrella term that encompasses augmented reality (AR), mixed reality (MR), virtual reality (VR), and other forms of alternative, expanded, or immersive reality applications. These and other forms of digital SBE have been shown to be an effective and cost-efficient method for training and are, therefore, now being used to replace or supplement training simulators and create learning scenarios beyond the classroom. Extended reality has been shown to be highly beneficial where visualization and interaction are required, training burdens are high, or material costs are expensive.

Historically, these digital tools were developed mainly by industry. However, as the entry to the market is lowered by decreasing costs and ease of use of these technologies, simulation technologists’ role pivots from that of the user to that of the developer. As a result of this shift, simulation technologists must acquire the additional skill sets needed to meet their evolving job descriptions and duties. This upskilling can be accomplished through traditional, focused courses offered by academic institutions and industry. However, participation in these courses may be perceived as a barrier, as they require considerable and sustained time commitments from the simulation technologists. An alternative approach is to provide these skills in a tailored, more distributed manner through micro-credentialing. Not only is this approach more feasible for busy technologists, but evidence from the field of educational science suggests that it is also a more practical approach.

Micro-credentials and the socio-technological movement

The omnipresence of the digital market and disruptive technologies has led to the acceleration of social change. In the higher education context, these socio-technological advances allow individuals to reskill, upskill, and upgrade their professional development. These advancements in technology, coupled with career shifts, are leading to changes in the education industry and innovations such as micro-credentials (MCs).

Micro-credentialing may be a transformative movement with the potential to disrupt current educational models. The need to be college/university- and career-ready implies that it is no longer sufficient for students to memorize facts, take standardized tests, and write term papers; they must work toward the mastery of specific competencies. MCs can support learners in this regard. They provide formal and systematic ways to develop and assess the competency acquired in an individual’s profession and support their progress and career advancement over time.

Micro-credentials are short and concentrated (hence “micro”) groups of personalized, competency-based courses that allow learners to earn proof of qualification (therefore “credentials”) by demonstrating mastery of the skills, knowledge, and behaviors required by the corporate, academic, and professional sectors. Since they are not time-limited, MCs offer flexible programming that deviates from traditional online courses or classes and is available at a lower cost. MCs verify, validate, and attest that specific competencies have been achieved to reach mastery. Industries and higher education organizations offer these courses to provide learners with the particular competencies that their work requires. The credentials earned by completing these courses can vary from degrees to diplomas, certifications, and digital badges. Although most of these forms of acknowledgment of credentials are similar to those found in traditional educational settings, digital badges are new and specific to micro-credentialing. These badges-visual representations of MCs granted once a learner has demonstrated mastery in the required skill-can be shared on social media, displayed on résumés, and inserted into a digital wallet.

The use of MCs is promising, as they offer the following outcomes: (a) the ability to revitalize career learning in a peer-driven manner and to improve educational outcomes; (b) the potential to bind both industry and higher education to provide career learning with demonstrated competency attainment; (c) the possibility of allowing individuals, including educators, to use professional learning to verify what they have achieved through competence attainment; (d) the low cost of MCs, which in conjunction with financial rewards, encourages individuals to participate in more career growth; (e) the potential of micro-credential and digital badges to increase higher education and student retention [2] and; (f) the possibility that using digital badging as a framework for demonstrating competency or mastery could contribute to additional educational developments. Digital badges can be used to provide course completion and learning evidence; establish micro-credentials; and represent excellence, participation, and community membership. Yet they can establish more than course completion and linear progression; they are granular, stackable, evidentiary, personalized, and machine-readable [3]. Further, MCs allow learners to learn or upskill, thus enabling their ability to stay current on specific topics. MCs are cost-effective and personalized to the individual learner’s needs; as such, they are evolving as a way to assess and evaluate individuals’ learning in their respective fields.

Local context

MCs began gaining momentum in Ontario with the launch by eCampusOntario, a provincially-funded non-profit organization that leads a consortium of the province’s publicly-funded colleges, universities, and indigenous institutes to develop and test online learning tools to advance the use of education technology and digital learning environments. Since 2017, eCampusOntario has led the initiative for the advancement and expansion of alternative learning through micro-credentialing-also known as micro-certification-through a shared structure and culture of experience. (Internationally, ISO certification standards are available to support MC design and implementation.)

The work of eCampus Ontario aims to improve and extend MC operation in Ontario. eCampus Ontario contributed to creating MC initiatives by establishing the Micro-Credential Principles and Framework [4]. The implementation and use of MCs align with core strategies outlined in eCampus’s overarching strategic plan: (a) model open and collaborative practices by supporting online teaching, curriculum, and tools for learners and institutions, (b) implement joint services to enhance learning and infrastructure while reducing costs, and (c) invest in research and development to create and evaluate innovative technologies and projects.

Higher education institutions, educators, learners, and policymakers must keep the lines of communication open and support career learning by focusing on learners’ achievement, participation, completion, and retention. In the prevailing socio-technological, cultural, and political context, novel collaborations between industry and post-secondary institutions represent a promising avenue for assessing MCs’ usefulness and efficacy: essentially, the industry can work closely with academia to ensure students graduate with the appropriate competencies, and, by employing MCs, these competencies can be updated quickly. A complement to traditional education, many companies, and postsecondary organizations have already begun to utilize MCs to provide a diversity of learning to benefit both students and employers. Such MCs can prepare individuals for employment by supporting them in developing human skills, intercultural competence, teamwork, and communication skills, among many others. Simulation-based activities have been used as part of micro-credentialing to provide experiential opportunities for learners to hone both interpersonal and technical skills. They may also be used to prepare individuals for a specific project; for example, MCs that provide skills that intersect with design, technology, and social theory could prepare workers to develop a new product prototype. These opportunities allow individuals to learn content that builds upon their confidence and interests and supports them as they navigate their careers after earning a degree or diploma.

For this editorial, we strictly focus on how SBE enhances MC’s ability to support and assess learning. Yet, we want to raise the issue of potential limitations of MCs as professional development-such as how instructors tap into the opportunities and support such as eCampus Ontario and how (if at all) the educational outcomes of MCs’ use benefit the diverse stakeholders. While this topic can be debated from many different perspectives and requires further investigation, it is beyond the scope of what is explored here.

Development of competence in health-professions education within the simulation context

Historically, competency has been measured by assessing learners’ clinical skills knowledge and attitudes (SKAs) rather than assessing their understanding of the science behind them. This perception has shifted over the past 40 years to consider not only one’s capabilities but also one’s ability to achieve desired objectives and to address and reflect upon clinical abilities that go beyond the performance of technical skills. This shift highlights the difference between a competent, a proficient, and a master worker.

A competent individual can select and execute actions appropriate to a given situation, whereas a skilled individual has the experience and ability to identify deviations to standard situations; knows the best approaches to solving problems; and can anticipate possible outcomes, concerns, and solutions. A master, however, has developed their own techniques to supplement what they have been taught and can extend their expertise within their domain. Thus, a skilled and competent individual is one who has developed their skills, supplements them with innovative techniques and problem-solving skills, and exhibits an ability to anticipate problems and intuit solutions.

Healthcare professionals’ education and training traditionally occurred in simulation labs within hospitals and higher education institutions, such as university-based medical and nursing programs. In these environments, learning can be customized and individualized and learners are provided with the learning path and objectives to allow them to progress from novice to master.

Simulation is not only an essential component of continuing education in healthcare but also an effective way to validate competencies in a controlled environment. One of the ways to assess competencies is by evaluating confidence. Confidence in one’s self-efficacy is a predictor of competent performance in new situations. Evidence shows that increased levels of self-efficacy are essential in skill acquisition, which is highly correlated with SBE.

Simulation-based education, therefore, provides a viable means for demonstrating and evaluating competencies consisting of elements such as critical thinking and interpersonal and technical skills. MCs require learners to demonstrate their competencies through either formative or summative assessments and evaluations; this, in turn, addresses the industry’s need to validate employees’ specific competencies. Simulation, therefore, can be an essential complement to MCs, as competency can not only be measured according to standards and protocols but also improved by development and training. As healthcare employers often prefer this type of validation for those seeking professional healthcare services positions, and as the competition among continuing-education providers increases, SBE is both a highly valued professional education tool and a significant differentiator. As such, we endorse simulation’s application in the shift to micro-credentialing.

Since MC courses are competency-focused, it is beneficial for learners to master their SKAs through SBE approaches. SBE methodologies, such as the use of simulated persons, for example, can provide learners with real-time feedback regarding areas to improve on and can allow them to master the SKAs in question. Simulation approaches provide teaching and learning opportunities to create, apply, and replicate real-world case scenarios; exposure to certain procedures through the use of technical and functional expertise training; access to simulated clinical or other workplace environments; and opportunities to enhance assessment techniques, teamwork, and workplace etiquette, and interpersonal communication, clinical judgment, leadership, and management skills, among many others. These teaching and learning opportunities also serve as a means for learners to demonstrate their competency to prospective employers. Since employability requires a combination of soft skills and core discipline skills that can be used on a day-to-day basis, these employability skills are transferable and can be adapted to different areas. Therefore, by incorporating SBE into MCs, postsecondary institutions can offer learners a mechanism for articulating to employers the competencies they gained throughout a program. Since employers are most interested in a candidate’s professional SKA, we recommend that higher education institutions use the content of this editorial to support a potential micro-credentialing structure that involves simulation for both competence development and assessment for their institution.

Using simulation to support competency assessment and skills within micro-credentialing contexts

Micro-credentials are awarded for evidence of competence. Learning pathways are scaffolded; that is, they are designed in such a way that learners progress towards a deeper understanding of content with the continued acquisition of digital badges or MCs [5]. When designing a learning pathway, clearly articulated and rigorous assessment methods and criteria are vital in ensuring the credential’s value. The assessment process demonstrates the application of SKAs and may involve industry professionals or the adoption of industry assessment standards.

MCs not only facilitate the learner’s progression from one level of competency to the next but also assess and validate the progression. Simulation supports both these abilities. High-fidelity simulators and assessment rubrics have demonstrated efficacy in assessing healthcare professionals’ ability to conduct a patient assessment; to use their clinical judgment, clinical reasoning, and communication skills; and to ensure patient safety. Conversely, low-fidelity simulations can be used to demonstrate proficiency in technical skills related to the practice. SBEs are, thus, a validated approach to illustrate one’s competency regarding foundational clinical skills and procedures. As such, the use of simulation-based approaches as the evaluation component for micro-credentialing can be considered a best practice for MCs; indeed, simulation must be integrated as part of the micro-credentialing frameworks used industry-wide. Examples of existing MC-associated courses include the Health Coach Professional Certificate and the Patient Navigator Professional Certificate, which are offered in Ontario.

To support the shift to MCs, it is critical that learning happens in small groups and is individualized. These are necessary both to engage learners and provide them with individualized feedback. Further, to embed simulation into MC delivery, there is a need to employ evidence-based strategies to support the practice, retention, and application of skills. This requires adequate practice, exposure, feedback on performance, and room for reflective practice.

As learners gain SKA and then learn ways of applying them, their competencies create foundational professional knowledge. Thus, it is crucial that we offer hands-on experience through means such as SBE early on in learners’ education journeys-even as early as high school. If, in addition, we implement micro-credentialing, these students’ MCs could be transportable to future educational settings and into the workforce.

Assessments for micro-credentials must include more than knowledge and understanding in the cognitive domain; instead, they should devise behavioral criteria that judge performance on a continuum of competence, reflecting industry or professional standards. Assessments should be authentic and generate evidence capable of reflecting the learner’s competence level. Simulation provides the appropriate methodology to support these types of assessment within micro-credentialing contexts. It is an essential tool for assessing beyond the cognitive domain; it can easily be mapped to competencies and supports feedback, recording, and reporting, all of which are inherent to the gold standard for micro-credential assessments. 

Conclusion

Healthcare professionals must possess specific competencies to perform their job requirements. SBE can identify what competencies learners may need to develop-including leadership, professionalism, communication and relation building, business skills, and understanding of the healthcare milieu-and enable learners to take steps to further their competency.

Effective competency-validation methods such as SBE require a dynamic process that includes educators and staff members and is dependent on the SKA to be assessed; the practice setting; and the learner’s expertise. The use of SBE in tandem with micro-credentials offers a two-fold advantage. First, MCs that incorporate simulation-based approaches provide an innovative means for healthcare educators and providers to acquire the competencies necessary to develop, implement, and evaluate effective teaching strategies and design curriculum-from online environments to practice settings. Second, MCs can support the measurement and advancement of competencies through SBE (including digital tools and disruptive technologies) to foster optimal learner outcomes.

Although we highly endorse the integration of SBE approaches in the creation and development of MC courses, we recommend that future research should include how simulation contributes to professional development when it involves micro-credentialing.
